# In vivo long-lasting alterations of central serotonin transporter activity and associated dopamine synthesis after acute repeated administration of methamphetamine

**DOI:** 10.1186/s13550-019-0557-y

**Published:** 2019-09-18

**Authors:** Wen-Sheng Huang, Guann-Juh Chen, Tung-Han Tsai, Chen-Yi Cheng, Chyng-Yann Shiue, Kuo-Hsing Ma, Skye Hsin-Hsien Yeh

**Affiliations:** 1 Department of Nuclear Medicine, Taipei Veterans General Hospital, No. 201, Sec. 2, Shipai Rd., Beitou District, Taipei City, 11217 Taiwan, Republic of China; 2 Department of Neurological Surgery, National Defense Medical Center, Tri-Service General Hospital, No. 325, Sec. 2, Chenggong Rd., Neihu District, Taipei City, 11490 Taiwan, Republic of China; 3 Department of Neurological Surgery, Chiayi Branch, Taichung Veterans General Hospital, No. 600, Sec. 2, Shixian Rd., West District, Chiayi City, 60090 Taiwan, Republic of China; 4 Nuclear Medicine Department, Tri-Service General Hospital, Taipei, Taiwan; 5 Department of Nuclear Medicine, National Taiwan University Hospital, No. 1, Changde St., Zhongzheng District, Taipei City, 10048 Taiwan, Republic of China; 6 Department of Anatomy and Biology, National Defense Medical Center, No. 161, Sec. 6, Minquan E. Rd., Neihu District, Taipei City, 11490 Taiwan, Republic of China; 7 Brain Research Center, National Yang-Ming University, No. 155, Sec. 2, Linong Street, Taipei City, 112 Taiwan, Republic of China

**Keywords:** 4-[^18^F]-ADAM PET, Methamphetamine, Long-lasting serotonergic neurotoxicity

## Abstract

**Background:**

Methamphetamine (METH)-associated alterations in the striatal dopamine (DA) system or dopamine transport (DAT) have been identified in clinical and preclinical studies with positron emission tomography (PET) imaging but have not been well correlated with in vivo serotonin transporter (SERT) availability due to the lack of appropriate imaging agents to assess SERTs. *N*,*N*-dimethyl-2-(2-amino-4-[^18^F]-fluorophenylthio) benzylamine (4-[^18^F]-ADAM) has been developed by our group and validated for its high affinity and selectivity for SERTs, allowing the in vivo examination of SERT density, location, and binding function. The aims of this study were to investigate the potential of SERT imaging using 4-[^18^F]-ADAM PET to estimate the long-lasting effects of METH-induced serotonergic neurotoxicity, and further determine whether a correlative relationship exists between SERT availability/activity and tyrosine hydroxylase (TH) activity in various brain regions due to the long-lasting consequences of METH treatment.

**Results:**

Male rats received four administrations of METH (5 or 10 mg/kg, s.c.) or saline (1 ml/kg, s.c.) at 1-h intervals. At 30 days post-administration, in vivo SERT availability and activity were measured by 4-[^18^F]ADAM PET imaging. In contrast to the controls, the uptake of 4-[^18^F]ADAM in METH-treated mice was significantly reduced in a dose-dependent manner in the midbrain, followed by the hypothalamus, thalamus, striatum, hippocampus, and frontal cortex. The regional effects of METH on TH activity were assessed by quantitative immunohistochemistry and presented as integrated optical density (IOD). A significant decrease in TH immunostaining and IOD ratios was seen in the caudate, putamen, nucleus accumbens, substantia nigra pars compacta, and substantia nigra pars reticulata in the METH-treated rats compared to controls.

**Conclusion:**

The present results suggested that the long-lasting response to METH decreased the uptake of 4-[^18^F]-ADAM and varied regionally along with TH immunoreactivity. In addition, 4-[^18^F]ADAM PET could be used to detect serotonergic neuron loss and to evaluate the severity of serotonergic neurotoxicity of METH.

## Background

The crucial role of the central serotoninergic system has been increasingly recognized in the field of addiction, as the reduction of serotonin transporters (SERTs) or upregulated serotonin levels contribute to the pathological mechanism and behavioral changes induced by drug abuse [1, 2]. The majority of pathological mechanism studies have focused on the effects of drug addiction in the striatum and amygdala, which are deeply involved in the development of addiction behaviors. Exposure to psychostimulants, as well as the withdrawal phase, is associated with gene-specific changes in SERTs in serotonergic terminals [3, 4].

The SERTs recycle serotonin to regulate its concentration in a gap, or synapse, and thus are the targets for selective serotonin reuptake inhibitors (SSRIs) [4]. While SERTs are mainly located on serotonergic terminals and cell bodies in the brainstem nuclei [5], they are also heterogeneously distributed in rat and human brains [6]. In rats, high densities of immunoreactivity were observed within the caudate, putamen, amygdaloid complex, cortical areas, substantia nigra, ventral pallidum, islands of Calleja, septal nuclei, interpeduncular nucleus, trigeminal motor nuclei, olfactory nuclei, and hippocampus [7]. In general, the binding of autoradiographic [^3^H]citalopram to its binding sites in SERTs in the human brain was highest in the limbic cortices, followed by the brainstem, striatum, pallidum, isocortex, and thalamus [8].

Neuroimaging using either positron emission tomography (PET) or single-photon emission computed tomography (SPECT) coupled with appropriate radiopharmaceuticals provides a noninvasive and functional means to evaluate drug effects on SERT distribution in the human brain. Imaging neurotoxic effects of 3,4-methylenedioxymethamphetamine (MDMA), an analog of methamphetamine (METH) that is known as ecstasy, has been extensively studied in nonhuman primates and humans using either [^123^I]β-CIT SPECT [9] or [^11^C]McN5652 and [^11^C]-DASB PET [10]. However, [^123^I]β-CIT and [^11^C]McN5652 are nonspecific for SERTs with only moderate signal contrast in human studies [9]. [^11^C]DASB is suitable for probing SERTs with PET; however, the short half-life of carbon-11 (~ 12 min) and the requirement of an on-site cyclotron hindered the possibility for routine practice in our laboratory. Because ^18^F has a half-life of 110 min, it can be produced off-site and transported to medical facilities for imaging use, and many ^18^F-labeled ligands have been developed for SERT PET [11].

Our group developed (*N*,*N*-dimethyl-2-(2-amino-4-[^18^F]-fluorophenylthio) benzylamine), termed as 4-[^18^F]ADAM, and demonstrated that, compared to others, 4-[^18^F]ADAM had suitable characteristics for imaging SERTs because of its high target-to-nontarget ratio, little in vivo de-fluorination, easy preparation, and acceptable radiochemical yield (~ 15%). Studies of toxicity and radiation dosimetry carried out in rats and rhesus macaques also suggested that 4-[^18^F]ADAM is safe [12]. The 4-[^18^F]ADAM PET and autoradiography for imaging SERTs have been validated in 5,7-dihydroxy tryptamine-lesioned and p-chloroamphetamine-induced, 5-hydroxy tryptamine (5-HT) depletion and paroxetine SSRI-treated rat models [12, 13] to evaluate the degrees of MDMA neurotoxicity and therapeutic response. We previously reported the neuroprotective effect of dextromethorphan against MDMA-induced neurotoxicity [14].

However, to date, few imaging studies have observed the in vivo changes in central SERTs in METH cases [15] but have focused on the alteration of dopamine or dopamine transport (DAT). Studies suggested that repeated high-dose treatment with METH in rats caused a decrease in central dopamine (DA) levels [16] and DAT binding [17]. METH causes significant depletion of serotonin [18], reduced tryptophan hydroxylase (TPH) activity [19], and decreased SERT binding [20] in rats.

Our group assessed the side effects of recreational drugs such as ketamine, cocaine, and METH on dopamine neurons in the peripheral organs using PET imaging and quantitative whole-body autoradiography with [^18^F]FDOPA, an analog of l-dihydroxyphenylalanine (L-DOPA). We demonstrated that the dose-dependent effect of acute administration (single injection) of these three recreational drugs and the inhibitory effects of the [^18^F]FDOPA accumulation (or the ability to raise dopamine) in the striatum or other tissues varied [21].

Repeated high-dose administrations of METH in monkeys also cause persistent decreases of dopamine and serotonin levels in the brain [22]. Studies in postmortem humans partially confirmed that METH abusers showed significantly reduced dopamine, TH, and DAT levels in the striatum and nucleus accumbens and decreased SERT levels [23]. Yamamoto and Zhu concluded that free radicals and oxidative stress, excitotoxicity, hyperthermia, neuroinflammatory responses, mitochondrial dysfunction, and endoplasmic reticulum stress might be responsible for the METH-induced neuronal fiber degeneration and apoptosis [24].

In a previous study, we demonstrated that lower 4-[^18^F]ADAM PET binding was associated with reduced SERT immunoreactivity by 6-hydroxydopamine (6-OHDA)-induced neurotoxicity in a rat model [25]. This result was in agreement with the study that reported by [^123^I]ADAM/SPECT imaging that SERT levels were decreased in monkey brains following 6-OHDA injections into the medial forebrain bundle [26]. Those studies determined that 4-[^18^F]ADAM PET could be used to detect serotonergic neuron loss or dysfunction of SERTs.

Based on the well-established experiences of SERT and DA imaging in animal models of substance abuse and addiction including MDMA, ketamine, cocaine, and METH, we modeled typical human METH exposure using acute administration of several repeated doses (5 or 10 mg/kg, s.c. four times, with each injection 1 h apart) in rats. The aims of this study were (1) to further characterize the long-lasting effects of METH by examining the brain region vulnerability and ligand binding to SERTs by 4-[^18^F]ADAM PET and (2) to determine the association of SERT availability/activity with the levels of TH, the rate-limiting enzyme for dopamine synthesis, and thus the maintenance of dopamine levels, used as a surrogate biomarker in METH-induced neurotoxicity.

## Materials and methods

### Animals and METH treatment

The animal study was performed according to the protocol approved by the Animal Care and Use Committee of the National Defense Medical Center Taipei, Taiwan (IACUC10-093). Male Sprague-Dawley (SD) adult rats (3 months old) weighing 250–300 g were housed in the animal center of the National Defense Medical Center at a constant temperature and a controlled 12/12-h light/dark cycle (light from 7:00 AM to 7:00 PM). Male rats were used to avoid the cyclic hormonal changes in female rats that are associated with the estrus cycle and could confound the results. METH was purchased from Sigma-Aldrich (St. Louis, MO, USA) and dissolved in 5 mg/mL saline (0.9% NaCl).

On the day of METH administration, rats were housed individually with restricted water and food. Rats received four administrations of METH (5 or 10 mg/kg, s.c.) at 1-h intervals or an equal volume of 0.9% saline [27]. After METH administration, two rats were housed per cage and had free access to water, food, and sawdust shavings.

According to previous studies [28], 30 days is adequate for the body to clear METH; however, the striatal DA levels are depleted to about 50% in METH-treated animals. Thus, the PET imaging of in vivo SERT availability/activity was carried out 30 days after the administration of METH to investigate the 30-day long-lasting depletion of serotonin as well as damage to striatal serotonergic nerve terminals. One week after the PET imaging study, the rats were sacrificed and tissues assessed by immunohistochemistry for TH levels.

### Synthesis of 4-[^18^F]ADAM

The 4-[^18^F]ADAM was synthesized in an automated synthesizer as described previously [29]. All the 4-[^18^F]ADAM formulations used in this study were prepared in our PET cGMP laboratory, which is inspected regularly by the Council of Atomic Energy and the Department of Health, Taiwan. The radiochemical purity was > 95%, and the specific activity was 0.6 Ci/μmol or 22.2 Gbq/μmol (EOB). All 4-[^18^F]ADAM was prepared in the PET-Cyclotron Laboratory of the National Defense Medical Center.

### 4-[^18^F]ADAM micro-PET imaging

Imaging protocols and acquisition for the METH-treated and vehicle groups (controls) were performed as described by Ma et al. [30]. Briefly, the rats were gas anesthetized (2% isoflurane with 98% oxygen mixture) and injected with 4-[^18^F]ADAM (14.8–18.5 MBq; 0.4–0.5 mCi) via the tail vein. PET imaging was performed 60–90 min after the administration of 4-[^18^F]ADAM.

The static PET images were acquired for 30 min on a small animal micro-PET R4 scanner (Concorde MicroSystems, Knoxville, TN, USA). The energy window was 350–650 keV, and the timing window was 6 ns. Images were then reconstructed by the Fourier rebinning algorithm and two-dimensional filtered back projection with a ramp filter with a cutoff using Nyquist frequency. The regional radioactivity concentration (KBq/cc) of 4-[^18^F]ADAM was estimated from the maximum pixel values within volumes of interest (VOI). The radioactivity concentration (KBq/cc, μCi/cc) was converted to percent injected dose per gram (%ID/g), and the mean and standard deviation values of radiotracer accumulation in various tissues were calculated. Specific uptake ratios (SURs) were expressed as (target region-cerebellum)/cerebellum [13].

Data were analyzed with *ASIPro* VM6.3.3.1 software (Concorde MicroSystem, Knoxville, TN, USA) or *PMOD* 3.7 software (PMOD Technologies Ltd., Zurich, Switzerland).

### Immunohistochemistry

Rats were anesthetized by intraperitoneal injection of 7% chloral hydrate (5 mL/kg). Their thoracic cavities were opened followed by an incision in the right auricle. Perfusion was performed via the ascending aorta with 300 mL of 0.9% normal saline followed by 300 mL of 4% paraformaldehyde in 0.1 M phosphate-buffered saline (PBS), pH 7.4. The rat brains were quickly removed and immersed in the same fixative for 2 h.

Then, the brain was stored overnight in a solution of 0.1 M PBS with 30% sucrose at 4 °C. The sagittal sections (30 μm) were cut using a cryostat (Leica CM 3050; Leica Microsystems Nussloch, GmbH, Nussloch, Germany). Afterwards, the brain sections were washed with PBS and incubated in 1% H_2_O_2_ in PBS for 30 min. Then, the sections were washed extensively with PBS and incubated to reduce background in blocking solution (1% normal goat serum in 0.1 M PBS plus 1% Triton X-100) for 1 h. Next, the sections were incubated overnight at 4 °C with rabbit anti-tyrosine hydroxylase (anti-TH) antibody (1:2000; Millipore Corporation, Bedford, MA, USA). Following the overnight incubation, the sections were washed and incubated with goat anti-rabbit biotinylated IgG (1:250; Vector Laboratories, Burlingame, CA, USA) for 1 h and with avidin-biotin complex (1:200; Vectastain ABC kit; Vector Laboratories, Burlingame, CA, USA) for 1 h. After these incubations, the sections were washed with PBS and exposed to 0.05% 3′3-diaminobenzidine (DAB, dissolved in 0.1% H_2_O_2_ in 0.05 M Tris buffer, pH 7.6) for 5 min. Finally, the sections were washed three times with distilled water and mounted on gelatin-coated glass slides.

IHC images were captured using an optic imaging system, Nikon OPTIPHOT-2 × 10 and MICROPHOT-FXA × 200–400 (Nikon Instruments Inc., Melville, NY, USA). The optical density of dopaminergic fiber images was analyzed and quantified with Image-Pro Plus v. 6.0 (Media Cybernetics, Inc., Rockville, MD, USA). The selected images were then converted into an 8-bit grayscale. The optical density was measured with the region of interest (ROI) and calibrated with a control region (corpus callosum). The ratio of ROI-to-control region was calculated and averaged for each animal.

### Statistics

Data are expressed as the mean ± standard deviation (SD). One-way analysis of variance (ANOVA) with post-hoc Bonferroni tests were used for statistical evaluations. A *p* < 0.05 was considered statistically significant. Statistical analyses of data were performed using GraphPad Prism 4 (GraphPad, La Jolla, CA, USA).

## Results

### Long-lasting effects of METH reduced regional serotonin transporter availability/activity

**Fig. 1:**
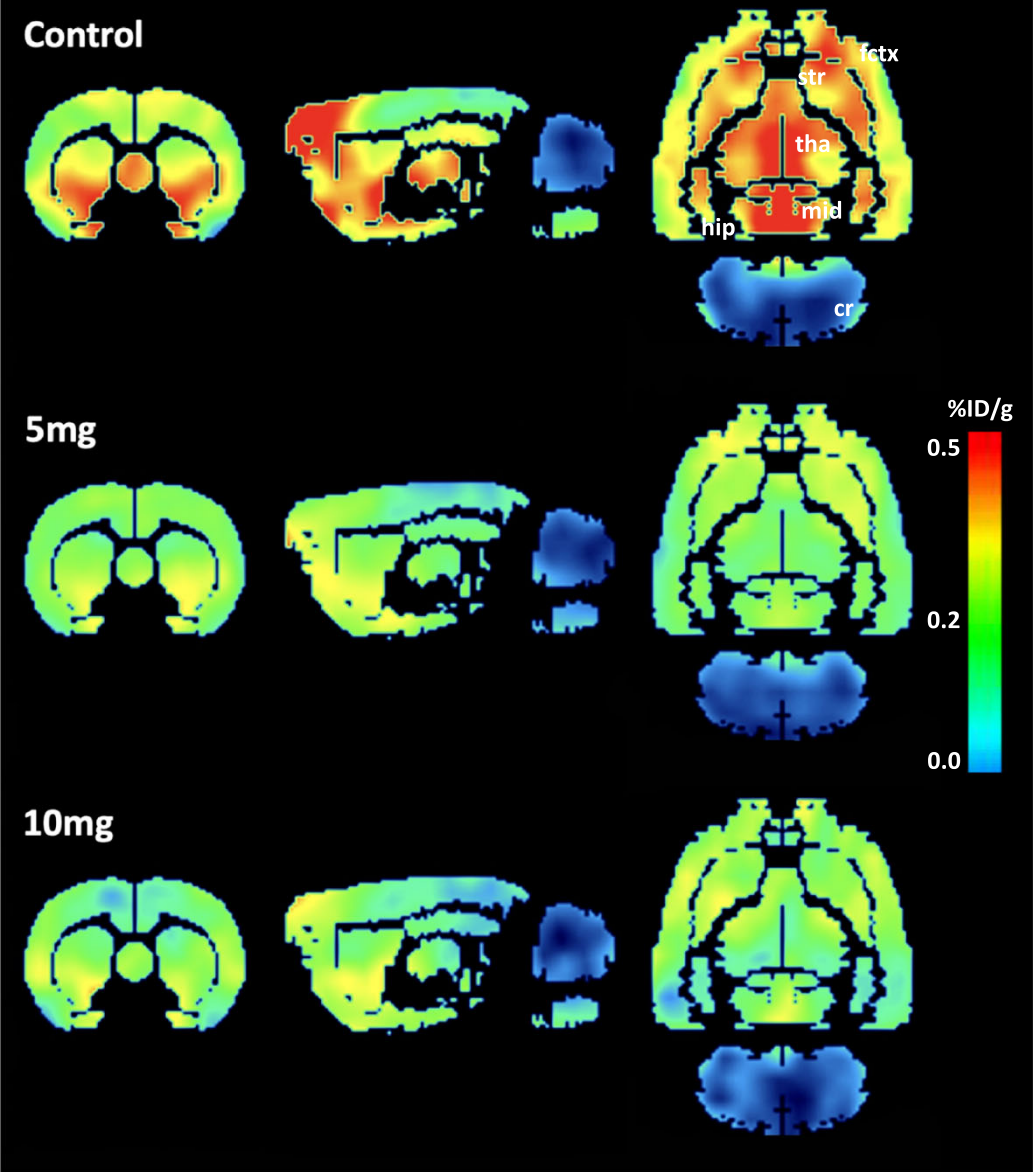
An example of 2D images showing 4-[^18^F]ADAM distribution obtained 60–90 min after administration of 4-[^18^F]ADAM in control animals (upper panel) and those treated with 5 mg/kg of METH (middle panel) and 10 mg/kg of METH (lower panel). Higher 4-[^18^F]ADAM accumulations were found in the amygdala, caudate/putamen, midbrain, and hippocampus. Lower accumulation of radioactivity was found in the cerebellum. The accumulation of 4-[^18^F]ADAM illustrates decreased SERT availability in the METH-induced groups. The accumulated radioactivity was expressed as a percentage of the injection dose/tissue gram (%ID/g). fctx, frontal cortex; str, striatum (caudate/putamen); tha, thalamus; mid, midbrain; hip, hippocampus; cr, cerebellum

**Fig. 2:**
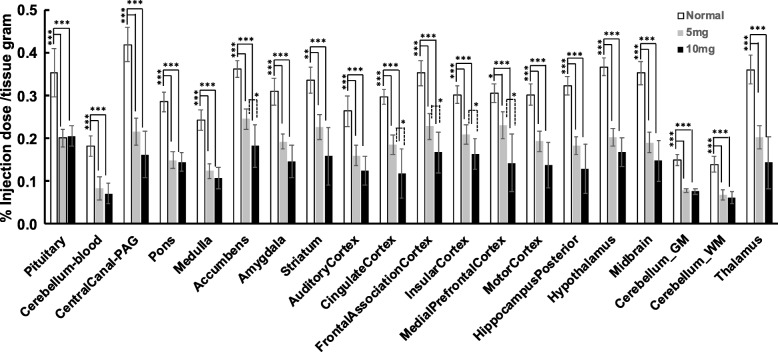
The accumulation of 4-[^18^F]ADAM, showing SERT availability, tended to decrease in most brain regions with increasing METH dose in rats. The higher dose of METH (10 mg/kg) caused a dramatic reduction of SERT availability. The accumulated radioactivity is expressed as a percentage of the injection dose/tissue gram (%ID/g) as the mean ± SD of 6–8 rats per group. *p < 0.05, **p < 0.01, and ***p < 0.001 compared to the control group

The distribution of radioactivity in normal rat brains at 60–90 min following intravenous injection of 4-[^18^F]ADAM as shown in Fig. 1 revealed increased 4-[^18^F]ADAM uptake in the SERT-rich regions, with the highest uptake in the periaqueductal gray matter (PAG), followed by the hypothalamus, accumbens, thalamus, pituitary, midbrain, frontal association cortex, striatum, hippocampus posterior, amygdala, medial prefrontal cortex, motor cortex, insular cortex, cingulate cortex, pons, auditory cortex, medulla, and cerebellum (Fig. 2). The results were consistent with our previous observations using the tissue counting method [29].

After 30 days, the long-lasting effects of METH were examined. The brain uptake of 4-[^18^F]ADAM was globally decreased, with the 10 mg/kg METH group having a greater decrease than the 5 mg/kg METH group, suggesting METH-induced SERT deficits might be dose dependent (Figs. 1 and 2). The uptake of METH in the brain was lower than that of other organs based on the unit of tissue volume (% injection dose/tissue grams, %ID/g). In addition, the clearance of 4-[^18^F]ADAM from the brain was relatively slow with a peak at 9 min and a half-peak clearance > 75 min after administration, resulting in a long-lasting brain exposure to its sympathomimetic effects [31].

**Fig. 3:**
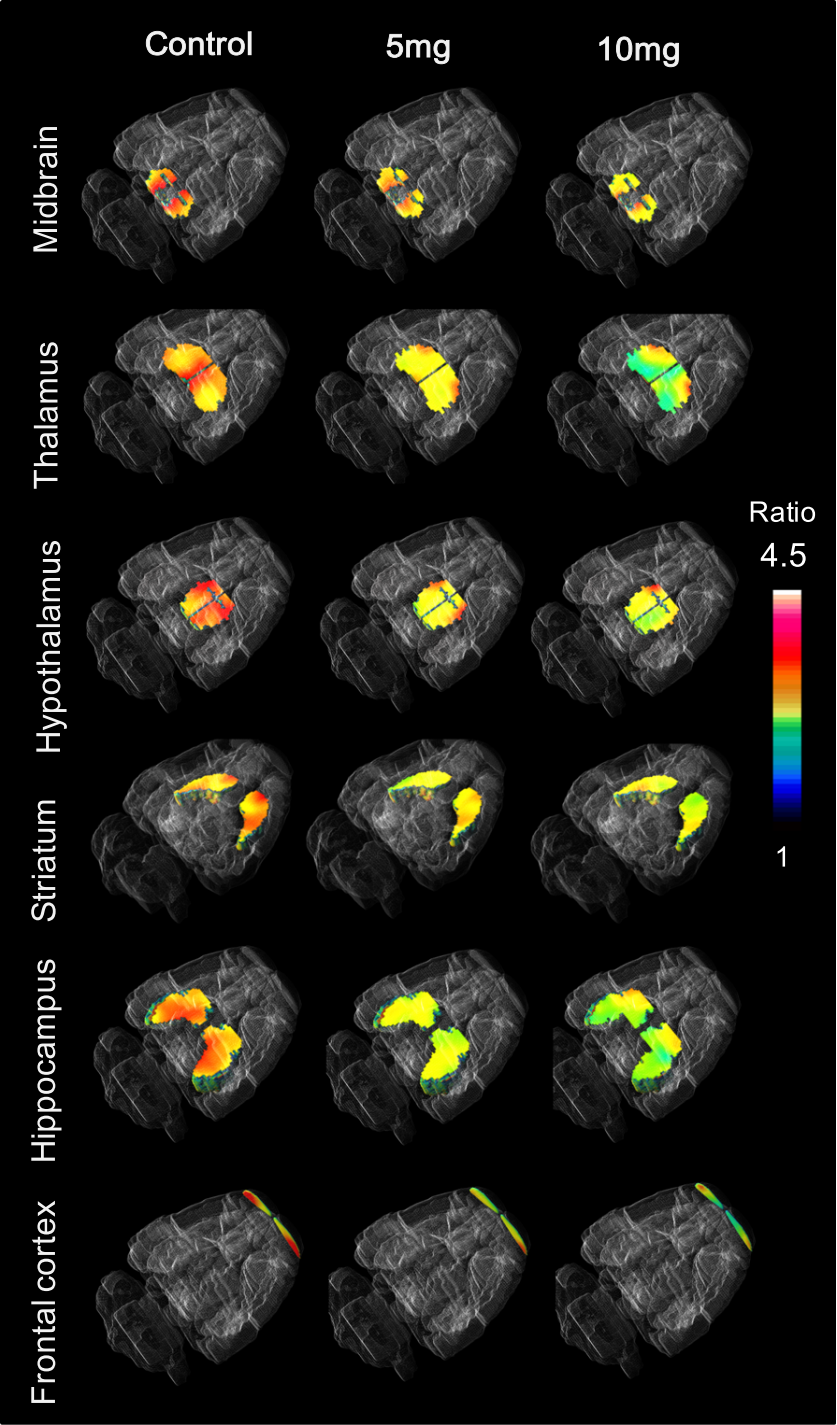
3D PET parametric images showing the specific uptake ratio (SUR) of 4-[^18^F]-ADAM in the brains of normal and methamphetamine-treated rats. The 10 mg/kg treatment group showed a globally lower 4-[^18^F]ADAM uptake in the brain compared to the 5 mg/kg group

**Fig. 4:**
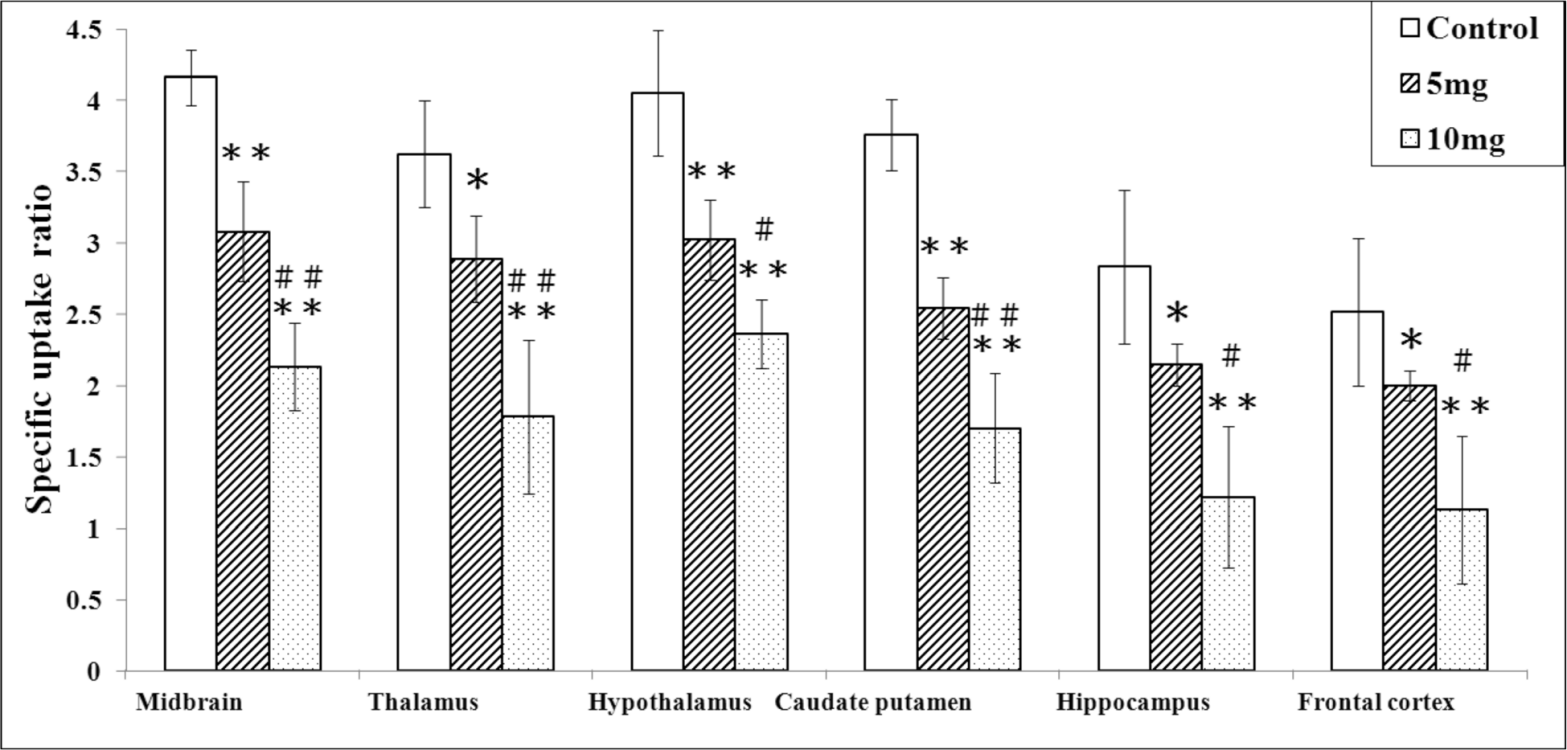
The specific uptake ratios (SURs) of 4-[^18^F]ADAM PET in various rat brain regions after repeated 5 or 10 mg/kg METH treatments. Data are expressed as the mean ± SD. *p < 0.05 and **p < 0.01 compared to the control group; ^#^*p* < 0.05 and ^##^*p* < 0.01 compared to the 5 mg/kg group; %, percentage of decrease compared to the control

The cerebellum is often used as a reference tissue in SERT imaging studies as a background value, to calculate the target-to-cerebellum ratio which is called the standard uptake ratio (SUR) [13]. The SUR PET parametric images showing 4-[^18^F]ADAM in both control and METH-treated rat brains at 60–90 min after 4-[^18^F]ADAM administration are illustrated in Figs. 3 and 4, respectively. In the controls, the uptake of 4-[^18^F]ADAM was found in SERT-rich regions: midbrain, hypothalamus, thalamus, and striatum. SUR analysis of each region yielded average SERT availability/activity of 3.83, 3.82, 3.34, and 3.21, respectively. There was an undetectable uptake in the cerebellum.

In the METH groups, the diminished uptake was more profound in the 10 mg/kg METH-treated group than in the 5 mg/kg group, with a decrease of 42.3 ± 1.3% and 86.6 ± 3.1%, respectively, compared to the controls (*p* < 0.01, Figs. 3 and 4).

### Dopamine synthesis was altered in METH-induced brains

**Fig. 5:**
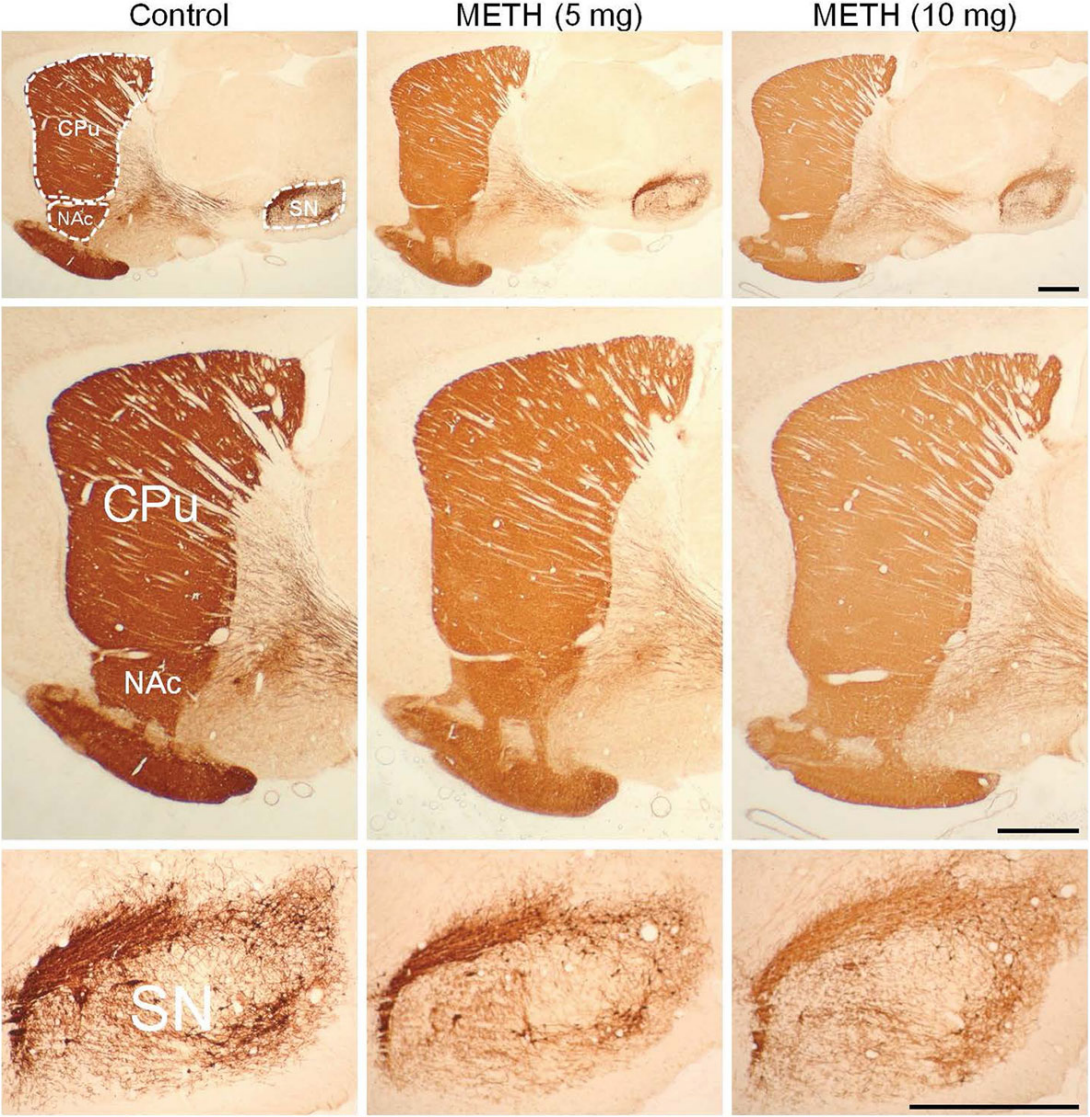
Immunohistochemical photomicrographs of tyrosine hydroxylase (TH) immunoreactivity at the para-sagittal levels of the control and METH-induced brains (upper panel) and their magnifications (middle and lower panels). CPu, striatum; NAc, nucleus accumbens; SN, substantia nigra

**Fig. 6:**
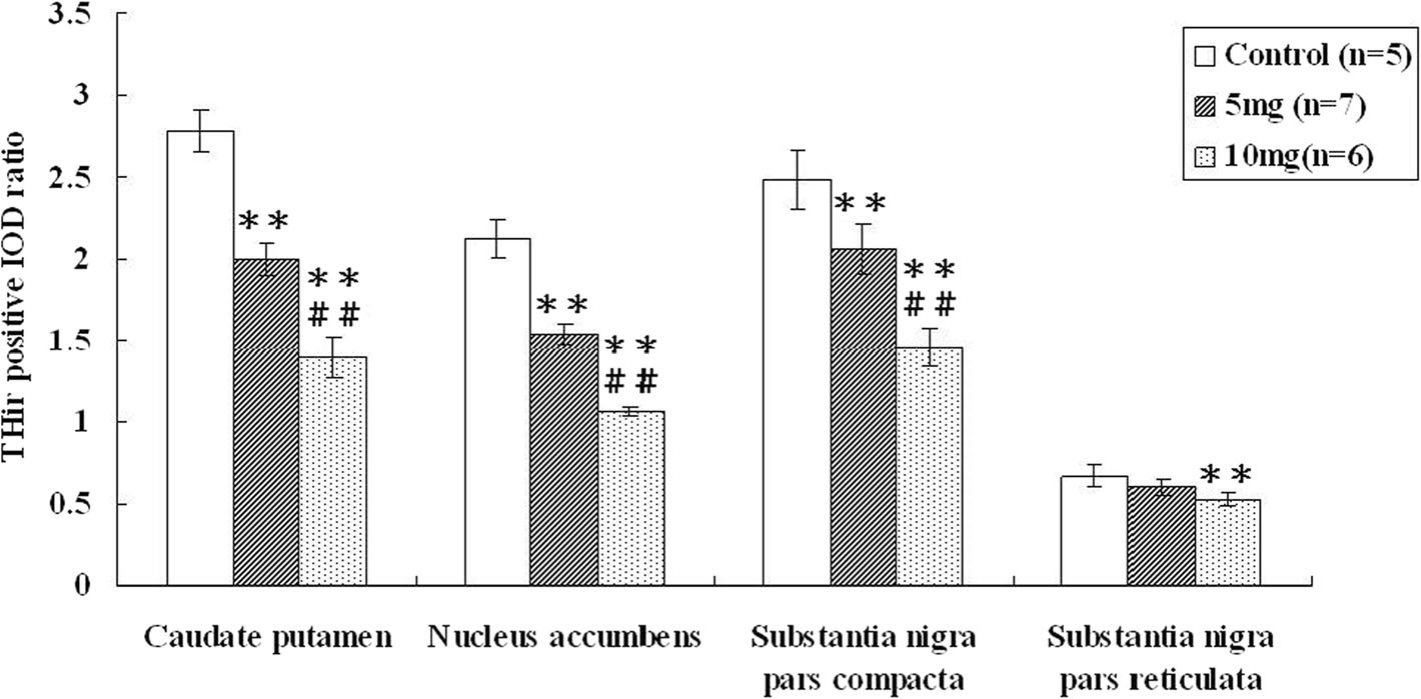
The integrated optical density (IOD) ratio of tyrosine hydroxylase (TH) immunoreactivity in the caudate-putamen (CP), nucleus accumbens (NA), substantia nigra pars compacta (SNPC), and pars reticulata (SNPR) of the control and METH-induced groups. *p < 0.05 and **p < 0.01 compared to the control group; ^#^*p* < 0.05 and ^##^*p* < 0.01 compared to the 5 mg/kg group

In addition to the SERT availability/activity measured by 4-[^18^F]ADAM PET, we further assessed the alterations in the cytoplasmic DA of METH-induced neurotoxicity. Immunocytochemistry results showed significantly decreased TH immunoreactivity in the caudate, putamen, nucleus accumbens, and, to a lesser extent, substantia nigra in the METH-induced groups compared to those in the control group (Fig. 5). Analyses of signal density found decreases of 21–50% in the OD ratios of the caudate, putamen, nucleus accumbens, and substantia nigra within the METH-induced brains relative to those of the control rats (*p < 0.01*) (Fig. 6). Although still dose dependent, the lower toxic effects of METH on the substantia nigra and decreased involvement of neuronal components in the pars reticulata compared with the pars compacta might be responsible for the differences for METH-induced toxicity (Table 1) [32].

**Table 1:** Comparable tyrosine hydroxylase immunoreactive positive cell numbers in the control and methamphetamine (METH)-treated rats’ substantia nigra

Region/group	Control (*n* = 5)	Methamphetamine dose (mg/kg)	
		5 mg/kg (*n* = 7)	10 mg/kg (*n* = 6)
Substantia nigra pars compacta	33 ± 1.29	33.24 ± 2.15	32.06 ± 2.26
Substantia nigra pars reticulata	4.73 ± 0.55	3.7 ± 0.62	4.11 ± 0.81

## Discussion

### Imaging SERTs with selected radiotracers for METH-induced neurotoxicity

The present data showed that the neurotoxic effects of METH led to alterations in levels of 4-[^18^F]ADAM binding in a dose-dependent manner. They also indicated that the high addictive potential of METH might be due to drug reinforcement related to its pharmacological effect of increasing dopamine in the nucleus accumbens [33]. The persistent dopamine excess due to repeated METH stimulation would eventually cause damage to the nucleus accumbens and other brain areas in the same way.

The present PET imaging and IHC findings, together with previous studies [16], implied that real-time in vivo 4-[^18^F]ADAM PET imaging might provide a reliable biomarker to observe the effects of drugs such as METH on brain function with minimal damage to the animals. This is especially important for primate studies and future clinical applications. There are various PET imaging radiotracers that can be used to examine the effects of drugs in serotonergic systems, for example, imaging dopamine D1/D2 receptors with [^11^C]PHNO, [^11^C]raclopride, or [^18^F]Fallypride [34, 35]; imaging DAT with [^11^C]PE2I [36, 37]; imaging synthesis of L-DOPA using [^18^F]DOPA [21]; and imaging SERT with [^11^C]DASB [38, 39] and [^11^C]McN5652 [40]. However, for SERT imaging, ^11^C-labeled [^11^C]DASB requires an on-site cyclotron for application and [^11^C]McN5652 has slow binding kinetics in the midbrain and high nonspecific binding.

### METH-induced changes in hydroxylase levels were associated with SERTs

Current results demonstrated that TH immunoreactivity in the caudate, putamen, nucleus accumbens, and substantia nigra was depleted after METH treatment. Our previous histological data support the association between TH immunoreactivity and brain uptake of 4-[^18^F]ADAM PET in a 6-OHDA-lesioned rat model [25]; however, in studies of METH-induced damage to serotonergic and dopaminergic systems, there is still a lack of direct comparisons between SERTs and DAT/dopamine synthesis PET imaging or between SERT and TH immunoreactivity. Thus, further investigation is needed into the differences in brain uptake between [^11^C]PE2I imaging of DAT [41] and [^18^F]FDOPA imaging of dopamine synthesis and 4-[^18^F]ADAM.

There is evidence to show that high doses of METH can cause loss of DA terminals, death of DA neurons, and TH-like immunostaining in the mouse olfactory bulb [41]. The authors also reported that METH-induced expression of activated caspase-3 in TH-positive cells was associated with increased expression of the proapoptotic proteins, Bax and Bid, but with decreased expression of the antideath protein, Bcl2 [42]. Park et al. further reported the expression of TH protein in the striatum, levels of TH mRNA, and the number of TH-positive neurons in the substantia nigra were reduced in the striatum after repeated METH treatment [43].

Taken together, these results suggested that METH-induced loss of DA terminals and death of DA neurons were, in part, via the mechanisms that were akin to an apoptotic process and reduced dopamine synthesis by decreasing the expression of TH.

In contrast to the striatum, where DA neuronal deficiencies were persistent, the alterations of TH immunoreactivity in the NAc explained the partial recovery at 14 days after the administration of METH and the resistance to METH-induced neurotoxicity, and its ability to recover revealed a fundamentally different neuroplasticity compared to the striatum [44]. Interestingly, our results in Fig. 5 showed that the 30-day long-lasting effect of METH caused a ~ 30% reduction in NAc TH immunoreactivity in the 5 mg/kg METH group (*p* < 0.01 compared to control), and the TH immunoreactivity fell to 50% in the 10 mg/kg METH group (*p* < 0.01 compared to control). Significantly, in the current experimental animal model, NAc did not show time-dependent recovery of TH.

Moreover, it has been reported that dopamine release in the nucleus accumbens (NAc) mediates the rewarding effects of METH; however, recent studies suggest that serotonin release may also contribute. Brown et al. [29] investigated the effects after 2 weeks of treatment with METH, and most serotonin axons in the dorsal striatum and NAc degenerated; however, the varicose axons in the shell appeared intact. These METH-resistant serotonin axons that lack SERTs densely innervate the caudal one third of the accumbens shell, the same location where dopamine axons are spared after METH [28].

Our results showed that 4-[^18^F]ADAM was decreased by 52% in the 5 mg/kg group (*p* < 0.01) and 60% in the 10 mg/kg group (*p* < 0.01) in the midbrain where SERTs are abundant. Different toxic effects or recovery from damage by METH between the serotonergic and dopaminergic systems might be a cause [45]. Acute repeated administration of METH (four administrations at 1-h intervals) may lead to the normalization of neurotransmitter or transporter and receptor activity; however, this hypothesis requires further investigation to verify.

### Limitations of the study

First, in this study, we found that a regimen of METH administration to SD rats resulted in a 30-day, long-lasting dual dopaminergic/serotonergic neurotoxicity. However, the extent of serotonergic and dopaminergic neurotoxicity caused by METH depends on several factors, including (a) animal species used (mouse vs. rat), (b) the rat strain used, (c) dosage and frequency of drug administration, and (d) death rates, which may increase due to severe side effects such as the thermoregulatory effect and muscle cramps caused by the drug.

Second, due to the impermeability of [^18^F]FDOPA PET, a fluorinated form of L-DOPA, and [^99m^Tc]TRODAT-1 SPECT, targeting of DAT, to the brain-blood barrier of rodent, we used TH immunoreactivity as a surrogate biomarker to verify the METH-induced neurotoxicity as shown in Figs. 5 and 6. Furthermore, in terms of extrapolating animal model data to humans, directly comparing blood-brain barrier permeability between different species would be a major issue for drug/radiotracer development studies of the central nervous system [46]. Nevertheless, although it is not an easy task, it appears to be feasible in principle to reconstruct the functions of transporters/receptors in vivo and to estimate species differences in drug distribution to the brain.

Third, although the decreased brain uptake of 4-[^18^F]ADAM PET was associated with tissue TH immunoreactivity, no direct comparison with SERT immunoreactivity was done due to the unavailability of staining materials.

Fourth, only adult male rats older than 3 months were selected in this study to avoid the possible variation of estrogen protection and age-dependent neurotoxicity of METH [47, 48].

## Conclusion

This preclinical study showed that the severity of METH-induced loss of SERTs could be reflected by 4-[^18^F]ADAM PET imaging, suggesting that 4-[^18^F]ADAM PET can be a feasible imaging technique to evaluate central SERT changes in METH abusers.

Further work is warranted regarding the effects of SSRIs on SERT availability in response to METH administration that may be clinically important for treating METH abusers, which could further clarify the interplay between METH and the brain serotonergic system. Another future area of study could be to assay the changes at the gene or protein level, to further determine if clinical trials of 4-[^18^F]ADAM PET imaging with METH abusers of the serotonin system are useful, whether it be for SERTs, TPH, or serotonin receptors.

## Data Availability

The datasets used and/or analyzed during the current study are available from the corresponding authors on reasonable request.

## Abbreviations

4-[^18^F]ADAM: N,N-dimethyl-2-(2-amino-4-[^18^F]-fluorophenylthio) benzylamine
6-hydroxydopamine (6-OHDA) [^18^F]FDOPA: [^18^F]-l-dihydroxyphenylalanine
DA: Dopamine
DAB: 3′3-Diaminobenzidine
DAT: Dopamine transporter
IHC: Immunohistochemistry
IOD: Integrated optical density
MDMA: 3,4-Methylenedioxymethamphetamine
METH: Methamphetamine
NAc: Nucleus accumbens
PAG: Periaqueductal gray matter
PBS: Phosphate-buffered saline
PET: Positron emission tomography
ROI: Region of interest
SD: Sprague-Dawley
SERTs: Serotonin transporters
SPECT: Single-photon emission computed tomography
SSRIs: Selective serotonin reuptake inhibitors
SURs: Specific uptake ratios
TH: Tyrosine hydroxylase
TPH: Tryptophan hydroxylase
VOI: Volumes of interest
